# Culture-independent bacterial cell extraction from fluid milk and oat-based beverage for basic qualitative microscopy

**DOI:** 10.3168/jdsc.2022-0320

**Published:** 2023-01-02

**Authors:** Samuel J. Reichler, Alicia Orta-Ramirez, Nicole H. Martin, Martin Wiedmann

**Affiliations:** 1 Milk Quality Improvement Program, Department of Food Science, Cornell University, Ithaca, NY 14853; 2 School of Health Sciences Blanquerna, Ramon Llull University, 08025 Barcelona, Spain

## Abstract

Butterfat and protein complicate attempts to extract bacterial cells from milk by centrifugation for use in basic microscopy. Some types of bacteria preferentially separate into the butterfat layer upon centrifugation and are lost when this layer is discarded, and the action of bacterial protease enzymes can cause milk proteins to precipitate and partition into the centrifugal pellet. Butterfat and precipitated protein remaining in the centrifugal pellet along with the desired bacterial cells can confound the results of differential staining and microscopy. Oat- and other plant-based beverages, which are often manufactured by dairy processors on shared equipment, present similar hurdles to bacterial extraction and microscopic visualization because of the presence of oils, starch granules, and dietary fiber particles in these products. Herein we describe methods for centrifugal separation of bacterial cells for microscopy from unflavored milk, chocolate milk, and oat-based beverage. Cell suspensions prepared through these methods were used for phase-contrast microscopy, Gram staining, and viability staining. These techniques can be used to provide rapid, culture-independent diagnostic information when bacterial cells are expected to be present in high concentrations, as in the event of sporadic product spoilage or mass product spoilage incidents.

## Highlights

• Butterfat, protein, and other milk components inhibit rapid microscopic visualization of microbial cells.
• Our novel method extracts microbial cells from fluid milk and other beverages, allowing for qualitative characterization.
• Our method provides dairy stakeholders with a culture-independent tool for troubleshooting microbial contamination.

## Graphical Abstract

**Figure:**
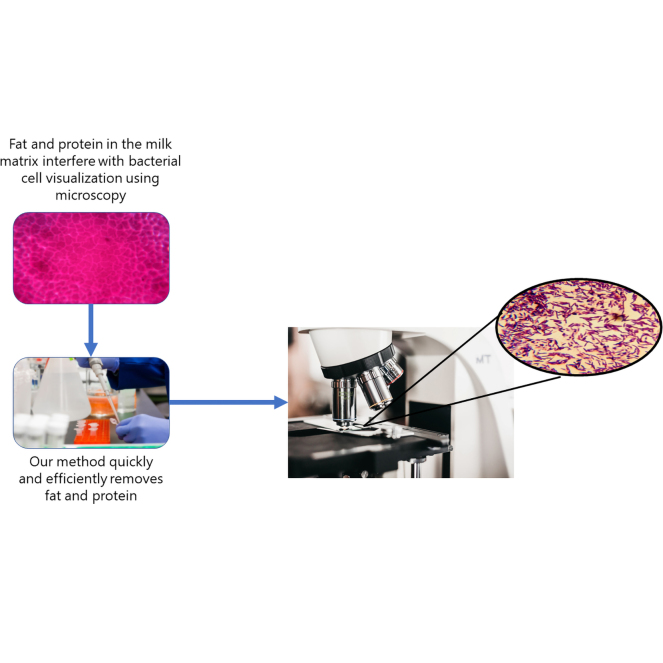
**Summary:** Microscopic techniques can provide rapid information for diagnosing microbial milk quality issues, but the butterfat and protein in milk can prevent microscopic visualization of bacteria. We describe a method to separate the bacterial cells in a milk sample from the protein and butterfat, allowing for better microscopic visualization. We adapted this method for use in chocolate milk and plant-based beverages as well. Use of this culture-independent method can reduce the amount of time needed to characterize the cause of bacterial spoilage, allowing for dairy stakeholders to respond more quickly to spoilage incidents.

Milk spoilage caused by heat-resistant gram-positive sporeforming bacteria and heat-labile gram-negative bacteria limits the shelf life of pasteurized fluid milk. The Gram status of the bacteria responsible for product spoilage provides critical information regarding the root cause of contamination and the appropriate focus for corrective actions. Crystal violet tetrazolium agar, the selective and differential medium most frequently used to identify total presumptive gram-negative bacteria from dairy products, requires 48 h of incubation and is unavailable for purchase in ready-to-use forms (Reichler et al., 2018). A Gram stain can also be performed from an isolated bacterial colony grown on nonselective agar-based plating media, such as that used for the standard plate count, also requiring 48 h for incubation. Culture-based tests for coliform bacteria can be completed in 24 h or less, but they exclude many types of gram-negative bacteria relevant to product quality and spoilage, including the genus *Pseudomonas* (Rojas et al., 2020).

Microscopy has an extensive history of use for rapid microbiological milk quality assessment. Direct microscopic bacterial clump counting is a well-established technique for initial culture-independent screening of raw milk quality, but it provides limited information on bacterial identity (Fitts et al., 2004). A previous attempt was made to apply the Gram stain method directly to prepared milk smears (Hucker, 1921). This technique requires solvent treatment of the dried milk smear to dissolve butterfat, a modified decolorizing solution, and an alternate counterstain, causing it to differ substantially from the contemporary standard Gram stain procedure (Beveridge, 2001). Both the direct microscopic bacterial clump count and the modified Gram stain of Hucker are limited by high detection thresholds and require the use of nonpolar solvents to achieve accurate staining and visualization of bacterial cells. To overcome the shortfalls of these 2 methods, we synthesized several previously described techniques to develop a new protocol allowing for rapid concentration and purification of the bacterial cells from unflavored and chocolate fluid milk samples. Our goal was to reduce the length of time needed to identify the Gram status of milk spoilage bacteria by allowing for culture-independent differential staining and microscopic examination of bacterial cells separated and concentrated directly from fluid milk. The key components of this method allow for protein solubilization and butterfat removal from a milk sample without considerable loss of bacterial cell integrity.

Precipitated protein is a barrier to the visualization of bacterial cells from fluid milk. We found that protein particles often partition into the centrifugal pellet, even in milk samples without visually apparent coagulated protein. These protein particles obstruct the view of bacterial cells when the resuspended pellet is observed using light or phase-contrast microscopy. They also retain the crystal violet used for the Gram stain, confounding the results of this test. Resolubilizing precipitated protein to clarify a milk sample is crucial for obtaining relatively pure preparations of bacterial cells from fluid milk. This is achieved by dissociating and solubilizing casein micelles, either by eliminating the hydrophobic bonds that stabilize casein micelle structure or by chelation of Ca^2+^ ions (Fox et al., 2015).

We assessed several previously described clarification solution formulations, including (1) 0.12 *M* EDTA, pH 8.0 (Brewster and Paul, 2016); (2) 0.1 *M* Bis-Tris, 8 *M* urea, and 1.3% trisodium citrate dihydrate, pH 7.0 (Visser et al., 1991); (3) same as (2) but with addition of 0.3 *M* 2-mercaptoethanol (Visser et al., 1991); and (4) same as (2) but with addition of 0.3% dithiothreitol, pH 8.0 (Miranda et al., 2004). We observed that although these solutions were very effective at resolubilizing protein, they resulted in visually reduced cell yields compared with unclarified samples. We ultimately selected a clarification solution described by Brown and Howe (1922) consisting of 0.6% wt/vol trisodium citrate dihydrate. In our observations, this solution produced acceptable levels of clarification while retaining the largest visible yield of intact bacterial cells. Furthermore, bacterial cells remained largely viable after treatment with 0.6% trisodium citrate.

Butterfat is an additional barrier to the visualization of bacterial cells from fluid milk for 2 reasons: (1) some types of bacterial cells, particularly sporeforming bacteria, partition with the cream layer rather than the pellet upon centrifugation of milk, confounding recovery efforts and result interpretation (Geer and Barbano, 2014); and (2) butterfat interferes with the staining and microscopy of bacterial cells because of its hydrophobicity. To eliminate butterfat from milk samples while preserving bacterial cell integrity, we selected a method described by Brewster and Paul (2016). Briefly, the milk sample is vigorously agitated in a horizontal shaker to force the bacteria that typically partition into the butterfat upon centrifugation into the aqueous phase of the milk. Following agitation, the sample is centrifuged and chilled to allow for physical removal of the butterfat. The resulting pellet is washed in a solution containing a nonionic surfactant to dissolve any residual fat. After butterfat removal and protein solubilization were performed, the resulting suspensions were found to be suitable for microscopy. The optimized method is described as follows.

Well-mixed milk samples (500 µL) were pipetted into 1.5-mL microcentrifuge tubes. Filter-sterilized 0.6% trisodium citrate dihydrate (Fisher Scientific) solution (1 mL) was added to each tube, and the tubes were vortexed briefly (approximately 5 s) to mix. Samples were incubated at 20 to 25°C for 1 h. The milk-citrate solutions were then heated to 40 ± 5°C and immediately placed into a horizontal microtube holder (Scientific Industries item no. SI-H524) attached to a benchtop vortex mixer. The samples were agitated at maximum speed for 10 min. Following agitation, the sample tubes were immediately transferred to a microcentrifuge (Eppendorf 5417C) and centrifuged at 5,000 × *g* for 5 min at 20°C to 25°C. This separated the milk into 3 phases: a pellet containing mostly bacterial cells and any remaining precipitated protein (bottom layer), skim milk (middle layer), and butterfat (top layer). The microcentrifuge tubes were chilled on ice for 10 min to solidify the butterfat layer. Sterile cotton swabs were used to remove and discard the butterfat layer. The supernatant (middle layer) was removed and discarded, and sterile cotton swabs were used to remove any visible butterfat remaining on the walls of the tube. Each pellet was then resuspended in 500 µL of PBS (Weber Scientific) containing 0.1% Tween-20 (Fisher Scientific) by pipetting vigorously and then vortex mixing thoroughly until the solution appeared homogeneous (approximately 5 to 30 s). The tubes containing the resuspended pellets were centrifuged at 5,000 × *g* for 5 min at 20°C to 25°C, the supernatant was discarded, and the wash procedure was repeated a second time. A third wash can optionally be performed before discarding the supernatant and resuspending the cell pellet in 20 µL of PBS (without Tween-20) for microscopy. The resulting suspensions should be examined immediately. Suspensions prepared according to this procedure were suitable for phase-contrast microscopy of a wet mount and brightfield microscopy of a Gram-stained smear (Figure 1). We did not specifically test bacterial extraction from raw milk using this method; however, it could be reasonably expected to perform similarly to pasteurized milk.

**Figure 1:**
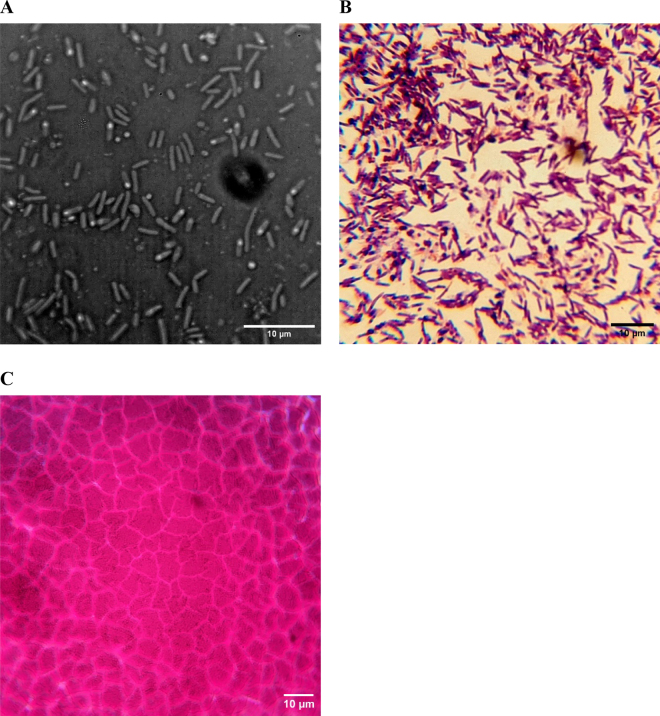
Photomicrographs of bacteria extracted per the protocol described here from a sample of 2% fat HTST pasteurized milk intentionally contaminated with *Paenibacillus odorifer* (isolate FSL J3-0155) using phase-contrast microscopy (A), Gram stain with brightfield microscopy (B), and Gram stain with brightfield microscopy without performing protein clarification (C).

This procedure can be adapted to chocolate milk. Bacterial extraction from chocolate milk is complicated by the presence of cocoa powder particles, whose size distribution overlaps with that of bacterial cells (Niediek, 1994). These particles partition into the pellet formed by centrifugation and prevent microscopic observation of the cells. A 2-step filtration process removes a sufficient quantity of larger cocoa particles to allow for successful microscopy. First, the sample is gravity filtered through a small plug of sterile cotton wool in a Pasteur or serological pipette. Second, the sample is gravity filtered through qualitative crepe filter paper with a particle retention size of 25 µm (VWR grade 415). After filtration, bacterial cells are extracted from the sample per the protocol for unflavored milk. The resulting suspension was found to be suitable for phase-contrast microscopy and Gram stain with brightfield microscopy.

Plant-based beverages are often produced and packaged by dairy processors on shared equipment. As is the case with dairy products, plant-based beverages can experience bacterial spoilage caused by contaminated raw ingredients and processing equipment. Bacterial extraction from plant-based beverages presents unique challenges not encountered in bovine milk. In addition to oils and protein, plant-based beverages, such as those prepared from starch-hydrolyzed oats, may still contain substantial quantities of starch granules and dietary fiber particles that can interfere with microscopy. We were able to extract nonviable bacterial cells from a commercially sterile, aseptically packaged oat-based beverage with incipient spoilage that appeared to have been caused by growth of *Bacillus*. A combination of enzymatic digestion, centrifugation, and filtration allowed for microscopic visualization of bacterial cells.

Oat-based beverage (250 mL) was aliquoted into a 250-mL polypropylene centrifuge bottle (Nalgene), and the contents of 2 commercial digestive enzyme capsules (Solgar) were added. The sample was shaken vigorously for approximately 30 s and then incubated at 37 ± 1°C in a shaking water bath (60 rpm). After incubation, the sample was immediately centrifuged for 5 min at 10,000 × *g* and 25°C. The supernatant was decanted and discarded, and as much fat as possible was removed with sterile spatulas and cotton swabs. The pellet was then resuspended by shaking in 100 mL of 0.85% saline with 0.1% vol/vol Tween-20. Centrifugation was repeated as in the previous step, the supernatant was discarded, and the resulting pellet was resuspended by shaking in 100 mL of 0.85% saline without Tween-20. This suspension was divided in half, and each half was gravity filtered through a fluted paper coffee filter. Both halves were recombined and centrifuged again (10,000 × *g*, 5 min, 25°C) with the resulting pellet resuspended by shaking in 5 mL of 0.85% saline without Tween-20. This suspension was divided into quarters, and each quarter was gravity filtered through qualitative crepe filter paper with a particle retention size of 25 µm (VWR grade 415). The recombined quarters were centrifuged again (10,000 × *g*, 5 min, 25°C), and the resulting pellet was resuspended in 30 µL of 0.85% saline for microscopy. This suspension was found to be suitable for phase-contrast microscopy (Figure 2A), Gram stain with light microscopy (Figure 2B), and viability staining with epifluorescence microscopy (BacLight Bacterial Viability Kit, ThermoFisher Scientific; Figure 2C).

**Figure 2:**
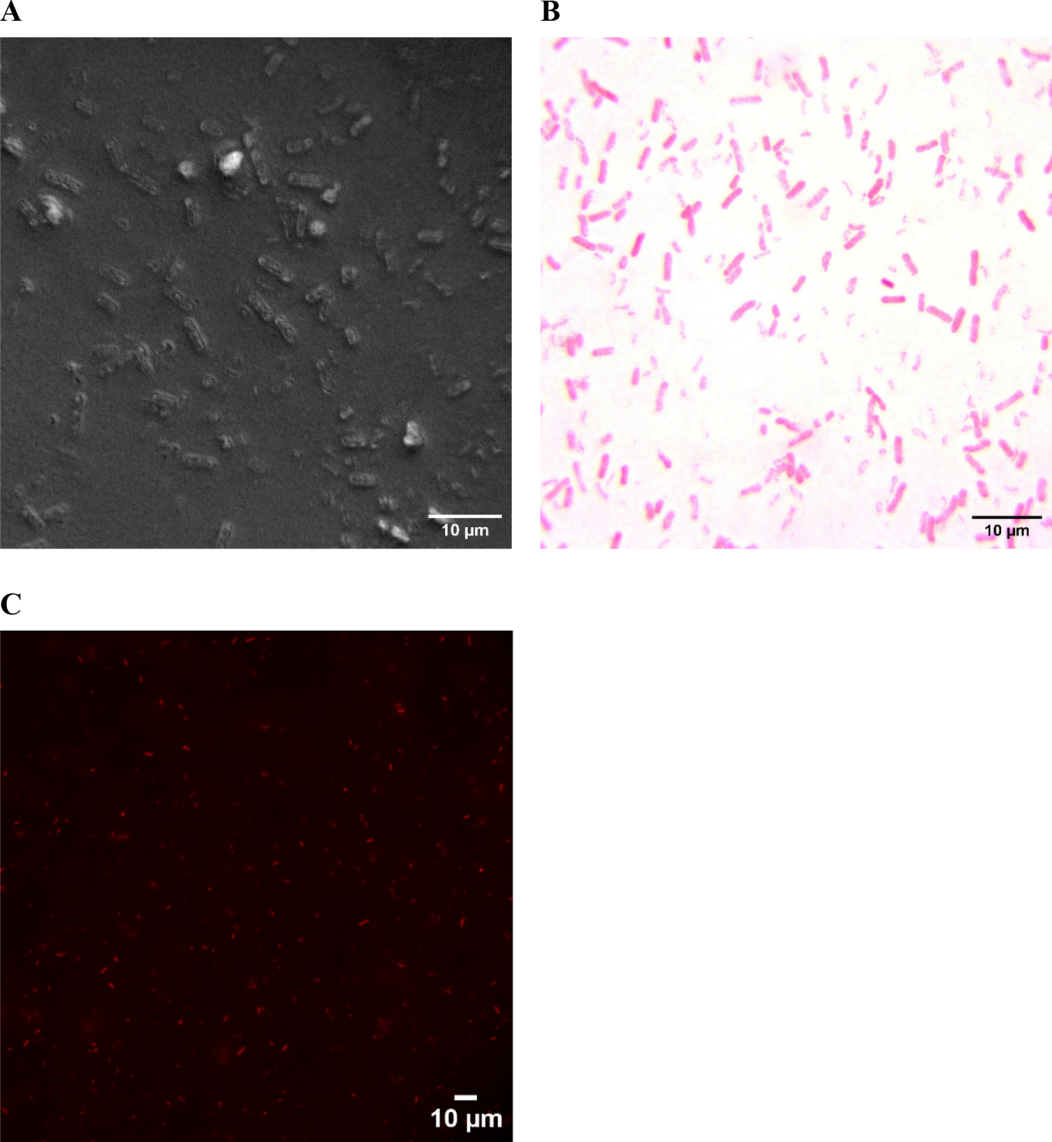
Photomicrographs using phase-contrast (A), Gram stain with brightfield microscopy (B), and viability stain with epifluorescence microscopy (C) of nonviable bacteria extracted per the protocol described here from a commercially sterile sample of aseptically packaged oat beverage with incipient spoilage resulting from unregulated growth of *Bacillus*.

Overall, the cell extraction method described here provides a rapid qualitative tool for use in the dairy industry; however, several limitations exist. For example, these methods are targeted toward products with high bacterial levels, such as those attained during product spoilage over refrigerated shelf life. These methods are not capable of detecting low-level contamination, including contamination with low levels of bacterial endospores. Further, these methods will extract both viable and nonviable cells from a sample, meaning that the methods cannot be used by themselves to determine cell viability. We demonstrated that viability staining can be performed successfully after the extraction. Despite the limitations, these protocols can be validated for specific products and microscopy procedures by inoculation of well-characterized bacterial type strains into sterile product samples. It may be possible to adapt these protocols to allow for approximate enumeration of bacterial cells using a counting chamber compatible with phase-contrast microscopy. These novel methods will provide processors with culture-independent diagnostic information in a matter of hours rather than days, allowing for faster and better-informed implementation of appropriate corrective actions.
